# Measles in Scotland

**Published:** 1915-11

**Authors:** 


**Measles in Scotland.** In 1913, 1,329 deaths from this disease occurred, the mortality being 2.8:1000. With 16 exceptions all deaths were of children under 15. (Caledonian Med. Jour.)

